# Should We Keep Walking along the Trail for Pancreatic Cancer Treatment? Revisiting TNF-Related Apoptosis-Inducing Ligand for Anticancer Therapy

**DOI:** 10.3390/cancers10030077

**Published:** 2018-03-18

**Authors:** Anna-Laura Kretz, Silvia von Karstedt, Andreas Hillenbrand, Doris Henne-Bruns, Uwe Knippschild, Anna Trauzold, Johannes Lemke

**Affiliations:** 1 Department of General and Visceral Surgery, Ulm University Hospital, Albert-Einstein-Allee 23, 89081 Ulm, Germany; anna-laura.kretz@uni-ulm.de (A.-L.K.); andreas.hillenbrand@uniklinik-ulm.de (A.H.); doris.henne-bruns@uniklinik-ulm.de (D.H.-B.); uwe.knippschild@uniklinik-ulm.de (U.K.); 2 Department of Translational Genomics, University Hospital Cologne, Weyertal 115b, 50931 Cologne, Germany; s.vonkarstedt@uni-koeln.de; 3 Cologne Excellence Cluster on Cellular Stress Response in Aging-Associated Diseases (CECAD), University of Cologne, Joseph-Stelzmann Straße 26, 50931 Cologne, Germany; 4 Institute for Experimental Cancer Research, University of Kiel, 24105 Kiel, Germany; atrauzold@email.uni-kiel.de; 5 Clinic for General Surgery, Visceral, Thoracic, Transplantation and Pediatric Surgery, University Hospital Schleswig-Holstein, 24105 Kiel, Germany

**Keywords:** TRAIL, pancreatic adenocarcinoma, TRAIL-R agonists

## Abstract

Despite recent advances in oncology, diagnosis, and therapy, treatment of pancreatic ductal adenocarcinoma (PDAC) is still exceedingly challenging. PDAC remains the fourth leading cause of cancer-related deaths worldwide. Poor prognosis is due to the aggressive growth behavior with early invasion and distant metastasis, chemoresistance, and a current lack of adequate screening methods for early detection. Consequently, novel therapeutic approaches are urgently needed. Many hopes for cancer treatment have been placed in the death ligand tumor necrosis factor (TNF)-related apoptosis-inducing ligand (TRAIL) since it was reported to induce apoptosis selectively in tumor cells in vitro and in vivo. TRAIL triggers apoptosis through binding of the trans-membrane death receptors TRAIL receptor 1 (TRAIL-R1) also death receptor 4 (DR4) and TRAIL receptor 2 (TRAIL-R2) also death receptor 5 (DR5) thereby inducing the formation of the death-inducing signaling complex (DISC) and activation of the apoptotic cascade. Unlike chemotherapeutics, TRAIL was shown to be able to induce apoptosis in a p53-independent manner, making TRAIL a promising anticancer approach for p53-mutated tumors. These cancer-selective traits of TRAIL led to the development of TRAIL-R agonists, categorized into either recombinant variants of TRAIL or agonistic antibodies against TRAIL-R1 or TRAIL-R2. However, clinical trials making use of these agonists in various tumor entities including pancreatic cancer were disappointing so far. This is thought to be caused by TRAIL resistance of numerous primary tumor cells, an insufficient agonistic activity of the drug candidates tested, and a lack of suitable biomarkers for patient stratification. Nevertheless, recently gained knowledge on the biology of the TRAIL-TRAIL-R system might now provide the chance to overcome intrinsic or acquired resistance against TRAIL and TRAIL-R agonists. In this review, we summarize the status quo of clinical studies involving TRAIL-R agonists for the treatment of pancreatic cancer and critically discuss the suitability of utilizing the TRAIL-TRAIL-R system for successful treatment.

## 1. Introduction

Typical characteristics of cancers are self-sufficiency in growth signals, enhanced proliferative signaling, invasion, metastasis, angiogenesis, replicative immortality, and resistance to cell death [1,2]. Pancreatic ductal adenocarcinoma (PDAC) harbors all of these classical hallmarks. Reduced response to conventional cytotoxic therapies is the culprit responsible for tumor development and progression despite therapy [3,4]. A less conventional therapeutic approach for PDAC arises from the stimulation of extrinsic apoptosis by death ligands. Accordingly, appropriate expression of respective death receptors (DR) on the plasma membrane and a functional downstream signaling apparatus is required to successfully stimulate the extrinsic apoptosis cascade reviewed in [4,5,6]. tumor necrosis factor (TNF)-related apoptosis-inducing ligand (TRAIL/Apo2L) and its pro-apoptotic receptors TRAIL receptor 1 (TRAIL-R1) and 2 (TRAIL-R2) represent such a death ligand/receptor system. On the one hand, the TRAIL-TRAIL-R system is expressed by PDAC cells and, on the other hand, capable of activating apoptosis selectively in tumor cells by binding to its death receptors reviewed in [7].

### 1.1. Pancreatic Cancer

Adenocarcinomas of the pancreas are nowadays still a significant health problem accounting for some 367,000 newly diagnosed patients in 2015 a number that keeps rising [8,9]. Compared to other solid tumor entities, pancreatic cancer has the highest mortality rate and is the fourth leading cause of cancer-related deaths worldwide [9,10]. Despite recent advances in diagnosis and treatment of PDAC, prognosis remains very poor, with a 5-year overall survival below 7% and a median survival upon diagnosis of 6 months [10,11]. This is mainly due to the aggressive phenotype of pancreatic cancer cells with highly invasive and metastatic potential. The only chance for cure is surgical resection [12,13]. However, due to vague and unspecific symptoms and lack of biomarkers, in most cases (80%) diagnosis only occurs at a locally advanced/metastatic and accordingly incurable stage. For these patients, treatment remains palliative to potentially alleviate symptoms and limit tumor growth [11,14]. Of note, tumor resected patients with curative interventions also frequently develop local tumor recurrence and/or metachronous metastases reducing long-term survival and potential cure [15]. 

Since 1997, monotherapy with gemcitabine (Gemzar), a synthetic pyrimidine nucleoside prodrug, was considered as the gold standard of care for PDAC. The nucleoside is an analog of deoxycytidine and is transferred to its active form by phosphorylation within the cell, eventually preventing DNA synthesis by incorporation into DNA. The efficacy of the substance is marginal as median survival is only prolonged by weeks to months. [16,17]. Consequently, combinatorial treatment strategies with gemcitabine were investigated the following two decades, again with unsatisfactory outcomes. However, improved response to therapy could be eventually achieved applying either the FOLFIRINOX (FOL-folinic acid-leucovorin, F-5-fluorouracil (5-FU), IRIN-irinotecan, OX-oxaliplatin) regimen [18] or the gemcitabine-nab (nanoparticle albumin-bound)-paclitaxel approach [17,19,20,21]. The folinic acid leucovorin is a vitamin B derivative to reduce side effects of 5-FU, a pyrimidine analog which incorporates into the DNA molecule and avoids DNA synthesis [22,23]. Irinotecan acts as DNA topoisomerase I inhibitor, which stops uncoiling and duplication of DNA [24]. Oxaliplatin, a diaminocyclohexane platinum compound, forms platinum-DNA adducts which block DNA replication [25]. Nab-paclitaxel is an example of nanotechnological cancer therapy. In this formulation, paclitaxel, first isolated in 1971 from the Pacific yew and described as an antitumor drug [26] is linked to albumin nanoparticles thereby increasing the agent’s solubility. The microtubule-stabilizing activity of paclitaxel enhances microtubule polymerization and G_2_/M phase arrest of the cells [27,28]. The FOLFIRINOX regimen and gemcitabine-nab-paclitaxel approach are the two combinatorial regimens which are the present standard treatments for advanced PDAC patients with good performance statuses. However substantial and severe toxic effects are reported when applying these regimes [21,29,30]. Meanwhile, therapy benefit for patients is still unsatisfactory. Notably, owed to the intrinsic or acquired resistance to apoptosis, pancreatic tumor cells are widely insensitive to conventional radio- and chemotherapy, mainly narrowing their efficiency [4,15]. Since apoptosis resistance to cell death is a hallmark of PDAC, only research into responsible molecular mechanisms will provide new treatment avenues towards targeted therapy [1,4,31].

### 1.2. TRAIL and TRAIL Receptors

A more in-depth understanding of tumor biology and the interplay between tumor and immune system promotes research into ‘targeted’ cancer therapy. One step in this direction was the identification of TRAIL, also referred to as Apo-2 ligand (Apo2L) in the mid-1990s [32,33] and its ability to induce apoptosis selectively in cancer cells while sparing healthy cells [34,35]. 

TRAIL is a member of the TNF-superfamily (TNF-SF) with the ability to induce apoptosis in cells by engagement of the DRs TRAIL-R1/DR4 (also called APO-2 or TNFRSF10A) [36] and TRAIL-R2/DR5 (TNFRSF10B, TRICK2, KILLER) [37] at the plasma membrane. Physiological full-length TRAIL is a type II transmembrane protein, constitutively expressed in a variety of tissues [32,33]. For many years, only limited data existed about the responsible proteolytic enzyme(s) involved in TRAIL cleavage aiding the release of its soluble form. Then, the protease cathepsin E was identified to release the 24 kDa extracellular portion of the ligand from the cell surface [38]. Similar to most TNF family members, the ligand forms homotrimers engaging three receptors [39,40]. In 1999, the crystal structure of the extracellular domain of human TRAIL was described, revealing that TRAIL monomers are composed of two antiparallel β-sheets which form a β sandwich as the core structure. The interplay of the TRAIL monomer with the adjacent subunits generates a bell-shaped homotrimer [41]. In order to stabilize and presumably mediate solubility and bioactivity of the ligand, an internal zinc atom interacts noncovalently with three cysteine residues, one supplied from each ligand monomer [42]. The crystal structure of TRAIL interacting with TRAIL-R2 revealed the trimerization of the TRAIL-R upon binding of the TRAIL trimer [39]. In healthy adults, the constitutive plasma concentration of soluble TRAIL is about 100 pg mL^−1^. Interestingly, at this concentration TRAIL exhibits no apoptosis-inducing activity in most cell lines and its function is therefore unknown [43,44].

In addition to TRAIL-R1 and TRAIL-R2, TRAIL can bind the receptors TRAIL-R3 and TRAIL-R4 also referred to as decoy receptor 1 (DcR1) alternative TNFRSF10C, TRID, or LIT and decoy receptor 2 (DcR2) alternative TNFRSF10D, or TRUNDD, respectively, as well as to the soluble receptor osteoprotegerin (OPG, TNFRSF11B) [5,45,46,47,48]. TRAIL-R1 and TRAIL-R2 signal apoptosis via their conserved death domain (DD) motif [37,47,49,50], whereas the DcRs have been shown to negatively regulate TRAIL-induced apoptosis [reviewed in 47]. TRAIL-R3 is attached to the plasma membrane via a glycophosphatidyl (GPI) anchor but lacks an intracellular domain, whilst TRAIL-R4 contains a truncated intracellular DD [5,36,47,49,50]. Overexpression experiments displayed the inhibitory potential of TRAIL-R3 and TRAIL-R4 regarding apoptosis induction by ligand scavenging and/or interaction with the death receptors [51,52,53] and down-regulation of the decoy receptors sensitized to TRAIL-induced apoptosis [54,55]. OPG, a soluble receptor identified to bind TRAIL, was initially demonstrated to regulate osteoclastogenesis by interaction with the receptor activator of nuclear factor κB ligand (RANKL). Notably, under physiological conditions the affinity of TRAIL for OPG is significantly weaker compared to the other TRAIL receptors [56,57], suggesting that OPG might play a subordinate role in modulating TRAIL-signaling. This may hold true since TRAIL present at low concentrations rather favors high-affinity binding to TRAIL-R2. Overall, the death-inducing TRAIL-receptors show higher affinity for TRAIL than the decoy receptors [58]. Since TRAIL-R2 exhibits the highest affinity for TRAIL at 37 °C, TRAIL-R2 was initially expected to be the primary inducer of TRAIL-induced apoptosis. However, this principal concept of TRAIL-R2 is questionable since it was uncovered that TRAIL-R1 is the primary inducer of apoptosis in pancreatic cancer cells and chronic lymphocytic leukemia even though TRAIL-R2 is expressed [59,60,61,62]. Synthetic TRAIL-R2-specific TRAIL variants were more efficient than wild-type TRAIL in killing PDAC [63]. Moreover, other data suggest that in certain scenarios also TRAIL-R2 might be the preferred TRAIL-R, mediating apoptosis in PDAC [64]. TRAIL-R1 and TRAIL-R2 share high sequence homology to a high degree (58% identical) [36,65] and so far, it is still enigmatic why two death-inducing TRAIL-Rs are expressed in humans. Two splice variants of TRAIL-R2 have been identified (long and short form), which differ by 29 amino acids in the extracellular domain. Moreover, the predominant occurrence of the long isoform was reported, whereas the ratio of the two isoforms differs in a tissue-dependent manner [66].

In comparison to the human system, mice only express one receptor capable of inducing apoptosis termed mTRAIL-R (MK) with 43% and 49% sequence homology to human TRAIL-R1 and TRAIL-R2, respectively [67]. However, the decoy receptors mDcTRAIL-R1 (TNFRS23) and mDcTRAIL-R2 (TNFRS22) lack a DD and differ substantially in their amino acid sequence from their human counterparts. Furthermore, they are not able to induce apoptosis [68]. Noteworthy, affinity of human TRAIL to the murine receptors is weak. Conversely, murine TRAIL interacts strongly with the human receptors [69]. 

**Figure 1:**
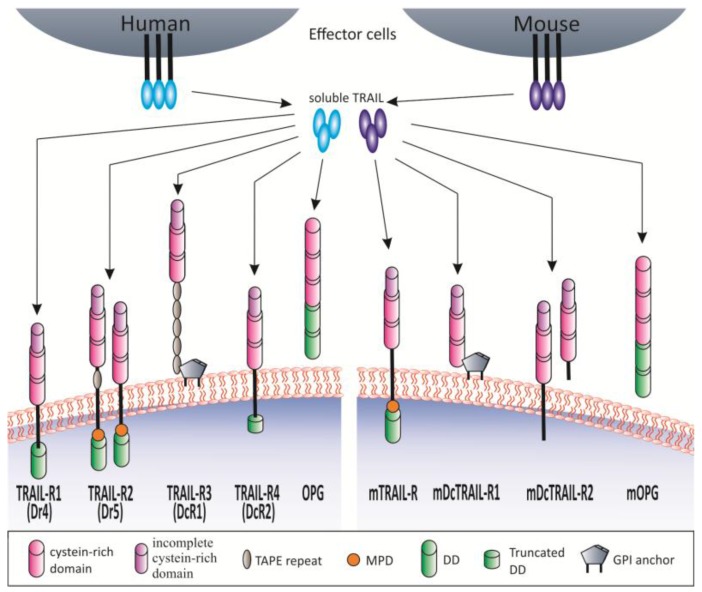
The human and murine tumor necrosis factor (TNF)-related apoptosis-inducing ligand (TRAIL)-TRAIL-receptor (TRAIL-R) system. TRAIL-TRAIL-R-system in humans. Human tumor-necrosis-factor-related apoptosis-inducing ligand (TRAIL) can engage four membrane-bound TRAIL-Rs and one soluble receptor, while two of these receptors can promote apoptosis (TRAIL-R1 and TRAIL-R2) and three might serve as decoys. TRAIL-R1 and TRAIL-2 express an intracellular domain comprising a death domain (DD), required for apoptosis induction. TRAIL-R2 occurs as short or long isoform, distinguishable by the absence or presence of a single TAPE (threonine/alanine/proline/glutamine) domain. TRAIL-R3 is attached to the membrane via a glycosylphosphatidylinositol (GPI) anchor and expresses five TAPE domains, but no intracellular domain. TRAIL-R4 merely expresses a truncated DD incapable of transmitting an apoptotic signal. Osteoprotegerin (OPG) represents the soluble receptor with low affinity for TRAIL. Cysteine-rich domains of the receptors are crucial for ligand binding. TRAIL-TRAIL-R-system in mice. Mice express four receptors for TRAIL, while mTRAIL-R is homologous to human TRAIL-R1 and TRAIL-R2. mDcTRAIL-R1 and mDc-TRAIL-R2 differ from the human forms. mOPG is the soluble receptor for TRAIL in the murine system. MPD, membrane-proximal domain.

A graphical representation of TRAIL and its receptors in humans and mice is presented in Figure 1.

### 1.3. Apoptotic Signaling

Apoptosis is a form of programmed cell death (PCD) and the central player in tissue homeostasis and embryogenesis first described in 1972 [70,71]. This cellular suicide mechanism designates the elimination of redundant, damaged, or infected cells by phagocytosis. It can be initiated either intrinsically via DNA damage, growth factor deprivation and cytotoxic stress and is in such cases termed intrinsic apoptosis or via extracellular death ligand binding to a death receptor (extrinsic apoptosis pathway). In all cases, apoptosis results in cell shrinkage, nuclear condensation, DNA fragmentation, and formation of apoptotic bodies. Caspases orchestrate these phenotypic hallmarks via cleavage of various target proteins [71,72,73]. 

**Figure 2:**
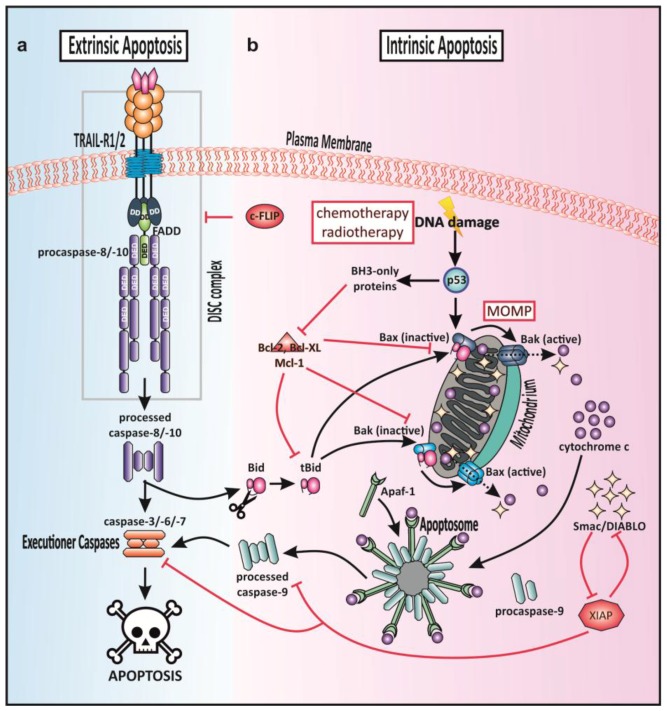
Apoptotic signaling. (**a**) Tumor necrosis factor-related apoptosis inducing ligand (TRAIL)-induced extrinsic apoptosis. Upon TRAIL binding to TRAIL receptor 1 (TRAIL-R1) and/or TRAIL receptor 2 (TRAIL-R2) the death-inducing signaling complex (DISC) is formed. In type I cells, the signal of DISC-activated caspase-8 is sufficient to activate downstream effector caspase-3 and therefore apoptosis, whereas the DISC signal needs amplification by mitochondrial apoptosis via caspase-8-dependent cleavage of the B cell lymphoma 2 (Bcl-2) homology domain 3 (BH3) interacting domain death agonist (Bid) in type II cells. Truncated Bid (tBid) translocates to the mitochondria and triggers the Bcl-2-family members Bcl-2-associated X protein (Bax) and Bcl-2 homologous antagonist killer (Bak) in the mitochondrial outer membrane (MOM), resulting in its permeabilization (MOMP) and eventually cytochrome c and second mitochondrial activator of caspases/direct inhibitor of apoptosis-binding protein with low pI (Smac/DIABLO) release. Apoptotic protease activating factor-1 (Apaf-1) complexes with cytochrome c and caspase-9 to build the apoptosome promoting effector caspases-3, -6 and -7. (**b**) Intrinsic death signaling. In response to stress signals, p53 becomes activated thereby triggering BH3-only proteins resulting in MOMP. In type I cells, DISC formation is sufficient to activate caspase-8 for activation of the executioner caspase-3. In type II cells, DISC formation is enhanced by MOMP to neutralize the caspase-inhibitory protein X-linked inhibitor of apoptosis protein (XIAP). XIAP binds to and potently inhibits caspase-3, -7 and -9. Several apoptosis regulation mechanisms are known: Fas-associated death domain (FADD)-like IL-1β-converting enzyme (FLICE)-inhibitory protein (c-FLIP) and caspase-8 compete for binding to FADD. Thus caspase-8 activation is repressed. In type II cells, binding of Bcl-2, B-cell lymphoma-extra-large (Bcl-XL) or induced myeloid leukemia cell differentiation protein (Mcl-1) to Bax and Bak, converts them into their inactive forms. Smac/DIABLO binds to and antagonizes XIAP and thereby ensures potent activation of caspases-3, -7 and -9.

Chemotherapy usually results in the induction of intrinsic apoptosis via p53 in response to DNA damage. The functionality of the tumor suppressor p53 is, therefore, a requirement for apoptosis induction. However, p53 is inactivated in around 70% of pancreatic tumors. Nevertheless, signaling through death receptors to induce apoptosis is still possible in many cases despite p53 inactivation [6,10,14,74,75,76,77]. In Figure 2, extrinsic and intrinsic apoptosis pathway are summarized. 

### 1.4. TRAIL-Mediated Cell Death Signaling—Extrinsic Apoptosis

The anti-tumor activity of TNF was first identified unknowingly when William B. Coley used dead bacteria (Coley’s toxins) to treat cancer at the end of the 19th century [78]. 100 years later, Old et al. demonstrated that this activity was attributable to a polypeptide, produced after bacterial infection. The hemorrhagic necrosis of treated tumors led to the polypeptide’s naming tumor necrosis factor in the 1970s [79]. TNF was enthusiastically debated as a novel potential anticancer substance for several years. However, it was revealed that canonical TNF-induced signaling triggers pro-inflammatory signaling pathways rather than apoptosis in most scenarios. Consequently, systemic application of TNF resulted in severe systemic inflammatory response syndrome reviewed in [80]. Novel hope was then placed in the discovery of the death receptor (DR) TNF superfamily receptor 6 (Fas)/apoptosis antigen 1 (APO-1) also called CD95 and the development of respective agonistic antibodies, reported to induce apoptosis in a variety of cancer cells [81,82,83,84]. Again, initial optimism was destroyed when fulminant and lethal hepatotoxicity was noted after systemic treatment with anti-Fas [85]. Subsequently, the sequence homology to TNF and Fas led to the identification and cloning of TRAIL [32,33]. Most striking this time, was the capability of TRAIL to selectively kill cancer cells in vivo without causing toxicity [34,35].

In response to TRAIL engaged with TRAIL-R1 and/or TRAIL-R2, extrinsic apoptosis is induced by the assembly of a signaling platform named the death-inducing signaling complex (DISC). In addition to TRAIL-Rs, this complex comprises the adaptor molecule Fas-associated death domain (FADD) and the initiator caspase-8/10 [86]. The exact stoichiometry of the TRAIL-DISC was uncovered in 2012 through quantitative mass spectrometry, revealing that three TRAIL-R1/R2 receptors recruit one FADD molecule which in turn recruits several procaspase-8 molecules [86]. The homotypic interaction of the death domain (DD) of FADD with the receptors respective DDs represents the bottleneck for activation of the TRAIL-mediated apoptosis pathway. FADD, in turn, enables the recruitment of procaspase-8 and -10 via homotypic interactions of their death-effector domains (DED) with the corresponding DED of FADD [3,45,86,87]. Since procaspase-8 contains two DEDs, a model proposed recently suggests that the first procaspase-8 molecule binds FADD directly, but all further procaspase-8 molecules bind to the free DEDs of the first procaspase-8 molecule in a growing chain [88]. DISC formation enables procaspase-8/-10 dimerization and proximity-induced activation required for initiation of the caspase cascade [3,45,86,87,88,89]. 

The caspase-8 homolog cellular FADD-like IL-1β-converting enzyme (FLICE)-inhibitory protein (c-FLIP) holds a crucial function in regulating caspase-8 activity within the DISC [90,91,92]. While this mechanism of regulation was initially understood to lie in a mere competition of c-FLIP and caspase-8 for binding to FADD, recent results support the idea that high levels of c-FLIP do not prevent direct caspase-8-FADD binding. The regulation rather occurs by suppressing elongation of caspase-8 chains and thereby their activation [93]. Due to alternative splicing, several c-FLIP variants exist, of which the most predominantly expressed ones are c-FLIP long (c-FLIP_L_) and c-FLIP short (c-FLIP_S_) [94,95]. c-FLIP_L_ and full-length procaspase-8 are strictly homologous. However, a catalytic cysteine present in the active site of caspase-8 and required for its proteolytic activity is absent in c-FLIP_L_. c-FLIP_L_ has been associated with both death-promoting as well as survival-promoting functions, depending on expression levels and strength of receptor stimulation [88,93,96]. c-FLIP_S_ comprises only DEDs [95,97] and was shown to act in an anti-apoptotic manner. 

In so-called type I cells, DISC formation is stable enough to sufficiently activate caspase-8 for efficient activation of the executioner caspase-3 resulting in apoptosis. In contrary, in type II cells, DISC-mediated initiation of the caspase cascade requires enhancement by mitochondrial outer membrane permeabilization (MOMP) to neutralize the caspase-inhibitory protein X-linked inhibitor of apoptosis protein (XIAP), a member of the inhibitor of apoptosis protein (IAP) family. XIAP binds to and potently inhibits caspase-3, -7 and -9 [98,99,100,101,102]. 

Pancreatic cancer cells have been classified as type II cells [103,104]. Consistently, members of the B cell lymphoma 2 (Bcl-2) family have been shown to crucially control extrinsic apoptosis in PDAC, as reviewed in [105]. The Bcl-2 family member Bcl-2 homology domain 3 (BH3) interacting domain death agonist (Bid) plays a major role in cross-signaling to mitochondria in response to DR activation. Upon DISC formation, active caspase-8 cleaves Bid generating truncated Bid (tBid), which translocates to mitochondria and eventually activates the mitochondrial cascade by triggering the oligomerization of the Bcl-2-family members Bcl-2-associated X protein (Bax) and Bcl-2 homologous antagonist killer (Bak), resulting in MOMP [45,106]. Following the pore formation in the mitochondrial membrane by Bak and Bax, the release of cytochrome c from the mitochondrial intermembrane space (IMS) to the cytosol is stimulated [107]. Once cytochrome c is present in the cytosol, apoptotic protease activating factor-1 (Apaf-1) triggers the assembly of the apoptosome, a stimulation platform for procaspase-9. Procaspase-9 holds an initiating function after activation through the apoptosome, promoting activation of effector caspases-3, -6 and -7 which execute apoptosis reviewed in [108]. 

Several regulation mechanisms are known which counteract excessive apoptosis. On DISC-level, c-FLIP and caspase-8 compete for binding to FADD. Thus caspase-8 activation is repressed and, consequently, execution of apoptosis [97]. In type II cells, binding of Bcl-2, B-cell lymphoma-extra-large (Bcl-XL) or induced myeloid leukemia cell differentiation protein (Mcl-1) to Bax and Bak, converts them into their inactive forms, thereby preventing MOMP [109,110]. Upon MOMP, in addition to cytochrome c, the second mitochondrial activator of caspases/direct inhibitor of apoptosis-binding protein with low pI (Smac/DIABLO) is released. Smac/DIABLO binds to and antagonizes XIAP and thereby ensures potent activation of caspases 3, 7, and 9 and execution of apoptosis [111,112]. 

### 1.5. TRAIL-R Agonists in Clinical Trials

In 1999, in vivo studies in mice, performed by two independent groups, revealed tumor regression after systemic treatment with recombinant variants of human TRAIL (rhTRAIL) [34,35]. This selective apoptosis-inducing effect of TRAIL on tumor cells while sparing healthy cells [34] provoked the clinical development of various TRAIL receptor-targeting agonists intended for cancer therapy. They can be subdivided into (i) recombinant human TRAIL variants and (ii) agonistic antibodies directed against TRAIL-R1 or TRAIL-R2 reviewed in [46]. Dulanermin (APO2L.0, AMG-951) is the only clinically available rhTRAIL ligand. The substance was not clinically evaluated for PDAC. Nevertheless, its suitability for this tumor entity will be revisited later with the aim to gain insights into the history of TRAIL-R agonist development. Only a few data are available on TRAIL-R-agonists in clinical trials for PDAC, these are the agonistic TRAIL-R2-specific antibodies conatumumab (AMG-655) and tigatuzumab (CS-1008). In the following section of this review article, these substances will be discussed in detail concerning their suitability for PDAC treatment.

### 1.6. Recombinant TRAIL

The challenge was to develop stable and active variants of recombinant TRAIL. Similar affinity for TRAIL-R1 and TRAIL-R2 might present a promising approach potentially enhancing death signals compared to stimulation of only one receptor. In this context, the death-inducing capacity might also be influenced by the different preference of TRAIL-R1 or TRAIL-R2 for apoptosis induction, varying tissue-dependently. Of note, in PDAC TRAIL-R1 is primarily used to trigger apoptotic cell death [59]. However, a potential problem regarding the therapeutic efficacy of recombinant TRAIL might arise from the fact that serum concentrations of active recombinant TRAIL available for TRAIL-R1 and -R2 binding might be downregulated by its binding to decoy receptors. In order to prolong half-life and stability of hrTRAIL, *N*-terminal tags such as poly-histidine tag [33], DYKDDDDK (FLAG) epitope [32] or leucine zipper (LZ) [34] motifs have been developed. Unfortunately, toxicology studies indicated that tagged recombinant TRAIL versions partially provoked hepatotoxicity, while this effect was absent with the use of non-tagged TRAIL [113].

To date, the only recombinant substance of human TRAIL approved for clinical use is dulanermin, an untagged variant based on amino acids 114–118 of the extracellular part of TRAIL [35]. Notably, dulanermin can bind both death-inducing receptors thereby providing a broader activity spectrum. However, the serum half-life of only 30–60 min actively restricts efficacy of the substance [114]. Dulanermin was clinically approved for non-small-cell lung cancer [115,116], non-Hodgkin’s lymphoma [117], metastatic colorectal cancer [118,119,120] and advanced cancer [114] in combination with chemotherapeutics. However, so far, in contrast to promising preclinical results [34,35], recombinant TRAIL failed as compared to standard therapy in clinical studies in the tumor entities mentioned. Although acceptable tolerability [114] was achieved in first in-human studies, significant antitumor effects were not observed as reviewed in [7,121].

### 1.7. TRAIL-R-Specific Agonistic Antibodies

Compared to recombinant forms of TRAIL, agonistic TRAIL-R-specific antibodies exhibit higher stability and prolonged half-lives. Conatumumab (AMG-655), a fully human agonistic monoclonal antibody (IgG1) directed against TRAIL-R2, was shown to induce apoptosis in vitro and in pancreatic cancer xenograft models in vivo. Furthermore, AMG-655’s activity cooperated with the antitumor potential of chemotherapeutics in PDAC in vivo models [122,123]. Following these encouraging preclinical results, phase I and II studies have been performed in patients with advanced PDAC [124,125,126]. AMG-655 was taken into a combination trial with gemcitabine in a phase Ib study in patients with metastatic pancreatic cancer to evaluate safety and efficacy. The study resulted in a disease control rate of 69% and a 6-month survival of 76.2% with acceptable tolerance in accordance with the first-in-human study [124,127]. Based on the results of the phase Ib study of AMG-655 combined with gemcitabine [124], a randomized placebo-controlled phase II study was performed in patients with metastatic pancreatic cancer comparing either ganitumab (AMG-479), a fully human anti-insulin-like growth factor receptor type I (IGF-1R) monoclonal antibody [128] or AMG-655 combined with gemcitabine. Trends were noted towards improved 6-month-survival. However, 12-month survival rate, overall survival, and patients’ response were not superior in comparison to the gemcitabine monotherapy Trends were noted towards improved 6-month-survival. However, 12-month survival rate, overall survival, and patients’ response were not superior in comparison to the gemcitabine monotherapy [126]. Tigatuzumab (CS-1008), the humanized form of the agonistic anti-TRAIL-R2 murine monoclonal antibody TRA-8, was made up of the complementarity-determining region of the murine mAb and the variable region framework and constant regions of human IgG-1 mAb58′Cl. As with the previously described mAbs, tigatuzumab convinced with promising preclinical results, including pancreatic cancer cell lines and murine PDAC xenografts [129,130,131,132,133,134]. In a phase I trial, tolerability was verified, and antitumor effects seemed to emerge, indicated by the high number of patients with stable disease [135]. A phase II study examining the combination of tigatuzumab and gemcitabine in patients with unresectable or metastatic pancreatic cancer was conducted [136]. In a nutshell, the results of the study were comparable to those of previous studies using gemcitabine monotherapy or in combination with other agents, given an overall survival of 8.2 months [136]. 

However, the underlying explanation for the clinical failure of TRAIL-R specific antibodies might be found in their mode of action. Since TRAIL engages TRAIL receptors as trimers, stimulation by bivalent antibodies results in stimulation of only two DRs and consequently in inadequate DISC formation reviewed in [46,137]. Moreover, it was uncovered that TRAIL-R1 becomes activated by membrane-bound TRAIL (memTRAIL) and soluble TRAIL (sTRAIL) whereas TRAIL-R2 is only activatable by memTRAIL or antibody-crosslinked sTRAIL [138]. Consequently, the capacity of TRAIL-R antibodies to induce apoptosis in pancreatic cancer might be feeble. In addition, tumor perfusion might represent a PDAC-specific problem [139,140,141]. Furthermore, the efficacy of recombinant antibodies directed against antigens on cancer cells relies on antibody-dependent, cell-mediated cytotoxicity (ADCC). Activatory and inhibitory fragment crystallizable (Fc)γ receptors (FcγRs) on immune cells regulate ADCC by binding to the Fc portion of immunoglobulins reviewed in [142]. The antitumor effect of the agonistic TRAIL-R2 antibody drozitumab was shown to be dependent on FcγR-mediated clustering [143]. These data suggest that for the general in vivo efficiency of agonistic TRAIL-R antibodies cross-linking is crucial. 

Potential interference of TRAIL-R-specific antibodies and endogenous TRAIL might also exist, competing for receptor binding. Intriguingly, dulanermin, the recombinant ligand, and AMG-655, both unsuccessful in clinical studies with the view of their antitumor efficiency, were demonstrated to synergize in killing TRAIL-resistant melanoma cells and also to increase antitumor activity in vivo. The combination of the substances provoked higher-order clustering of TRAIL-R2 thereby intensifying DISC formation [144,145]. Whether this effect also holds true for pancreatic cancer remains to be shown.

### 1.8. Dysfunctional TRAIL-Induced Apoptosis in Pancreatic Cancer

Even though anticancer effects of TRAIL in a variety of cancer cell lines has been demonstrated [34,35], many primary tumor cells are inherently resistant to TRAIL-induced apoptosis [146]. These cells exploit mechanisms to circumvent death ligand-mediated apoptosis [104,147,148], which are not entirely understood to date. The decision to live or die is taken at the level of pro and- antiapoptotic protein ratio in any cell. Notably, TRAIL-induced apoptosis can be inhibited at every step along the cascade by their respective anti-apoptotic proteins namely DcRs, c-FLIP-isoforms, and anti-apoptotic members of the Bcl-2-family such as Bcl-2 and Bcl-XL, and inhibitors of apoptosis proteins (IAPs) (Figure 2) [149]. Consequently, there are several possible mechanisms for tumor cells to harbor or acquire TRAIL resistance. PDAC cells exhibit resistance to TRAIL-induced apoptosis, primarily a result of constitutively up-regulated anti-apoptotic proteins such as c-FLIP, TNF receptor-associated factor 2 (TRAF2), Bcl-XL, and XIAP [103,150,151]. 

A study identified low expression of *O*-glycosylation (*O*-glyc) genes as further potent mechanism contributing to TRAIL resistance. In PDAC inter alia, a correlation of the peptidyl *O*-glycosyltransferase GALNT14 mRNA expression with TRAIL sensitivity attracted attention. *O*-glyc of TRAIL-R2 triggers ligand-mediated clustering of the receptors and consequent recruitment and activation of caspase-8 [152]. Recent results demonstrate the enhancing effects on TRAIL-induced apoptosis mediated by *N*-glycosylation (N-glyc) of TRAIL-R1 while TRAIL-R2 is deficient of *N*-glyc sites. Dysfunction of apoptotic signaling was connected with *N*-glyc-deficient TRAIL-R1 resulting in lower receptor aggregation and reduced DISC formation [153]. These data suggest a different promotion of apoptosis by these two receptors since TRAIL-R1 is *N*-glycosylated and TRAIL-R2 is *O*-glycosylated.

The capability to induce apoptosis is reserved for TRAIL-R1 and TRAIL-R2 [5,47,50]. An imbalance between expression levels of non-cell-death-inducing and cell death-inducing TRAIL-Rs might influence apoptosis signaling [51,52,53,54,55]. The truncated death domain of TRAIL-R4 induces nuclear factor kappa light chain enhancer of activated B cells (NF-κB), a transcription factor complicit in pro-inflammatory immune responses, presenting protection against apoptosis [104,154]. NF-κB was found to be constitutively active in pancreatic cancer cells resulting in elevated levels of XIAP thereby preventing apoptosis [104,155]. Expression of anti-apoptotic proteins such as TRAF2 and c-FLIP is also connected with the activation of NF-κB [156,157]. Furthermore, it was demonstrated that TRAIL-R4 forms heterotrimers with TRAIL-R2 to build ligand-independent inactive structures to prevent apoptosis or initiate gene-activating pathways such as NF-κB-mediated ones [45,154]. Death- and survival pathways can be switched in clonal populations of cancer cells in response to the treatment. The result is ‘fractional killing’ of the parental cellular population, while the surviving resistant fraction is selected. This ‘fractional killing’ by therapeutics is not only stem-cell dependent, however, significant variability can also arise from natural alterations in protein levels [158]. In this context, it was revealed that TRAIL-R2 plays an independent dual role in simultaneously mediated pro-apoptotic and pro-survival signaling [159]. 

### 1.9. TRAIL-Mediated Non-Canonical Signaling

**Figure 3:**
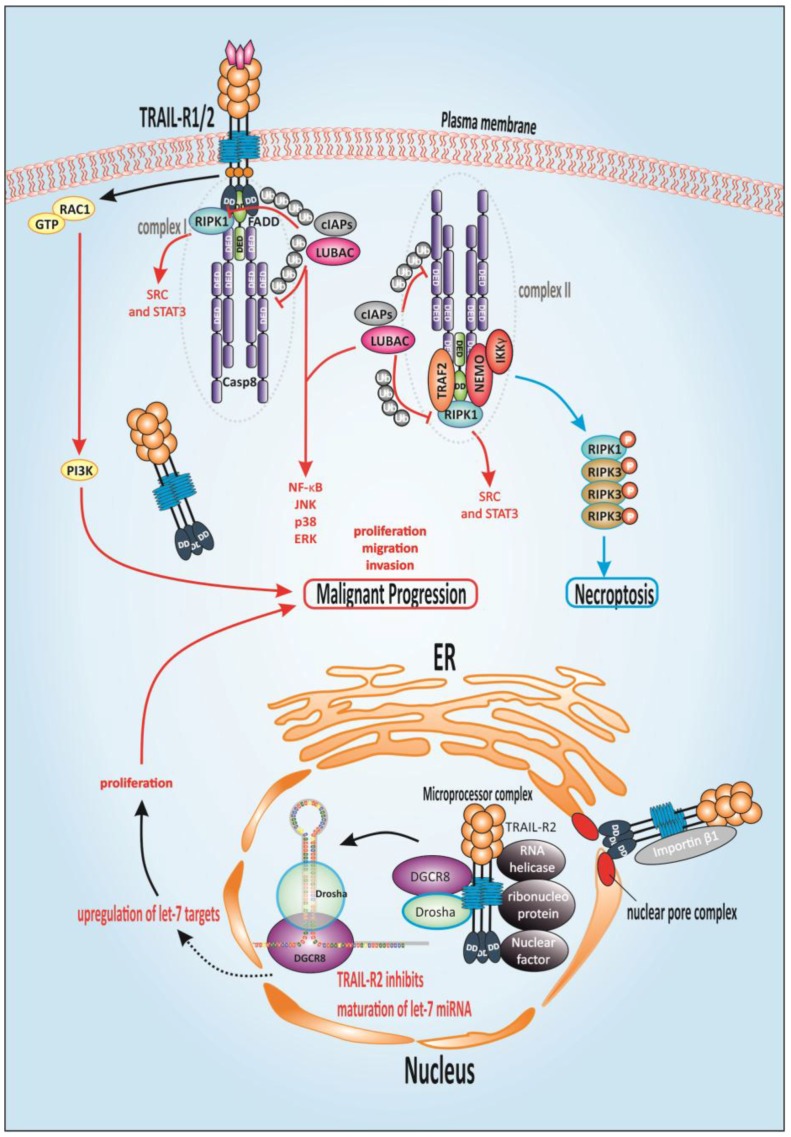
TRAIL-mediated non-apoptotic signaling. TRAIL binding induces assembly of a second cytosolic complex, retaining TNF superfamily receptor 6 (Fas)-associated death domain (FADD) and caspase-8 and recruiting receptor-interacting protein kinase 1 (RIPK1), tumor necrosis factor (TNF) receptor-associated factor 2 (TRAF2), and nuclear factor kappa light chain enhancer of activated B cells (NF-κB) essential modifier (NEMO). Downstream of caspase-8, TRAF2 recruits cellular inhibitor of apoptosis protein 1/2 (cIAP1/2) which in turn leads to the ubiquitination of RIPK1 and therefore recruitment linear ubiquitin chain assembly complex (LUBAC), that attaches linear poly-ubiquitin chains on RIPK1 [167]. RIPK1 is compulsory for the stimulation of tyrosine-protein kinase Rous sarcoma oncogene cellular homolog (SRC) and signal transducer and activator of transcription 3 (STAT3) which are responsible for pushing migration and invasion. Complex I and complex II trigger NF-κB, p38 mitogen-activated protein kinase (p38 MAPK), JUN *N*-terminal kinase (JNK) and extracellular signal-regulated kinase (ERK). LUBAC acts in both complexes, alleviating caspase-8 activation and enabling recruitment of the Inhibitor of κB (IκB) kinase (IKK) complex, and consequently activation of NF-κB. In case of inhibited caspase activation, the necrosome is built by the interaction of RIPK1 and RIPK3. Independently of FADD and complex I and II, the membrane-proximal domain (MPD) of TRAIL receptor 2 (TRAIL-R2) induces Rat sarcoma (Ras)-related C3 botulinum toxin substrate 1 (Rac1) activation to promote migration and invasion. TRAIL-R2 is also present in the nucleus and interacts with ribonucleoprotein complexes attributed to the maturation of microRNAs (miRNAs) of the let-7 family which interact with and inhibit mRNAs of various regulators of mitogenic pathways such as Ras and avian myelocytomatosis virus oncogene cellular homolog (c-Myc) thereby promoting proliferation. DD: death domain; DED: death effector domain; ER: endoplasmic reticulum; GTP: guanosine-5′-triphosphate; Ub: ubiquitin; DGCR8: DiGeorge critical region 8; P: phosphorylated.

In recent years, it has become evident that above and beyond the classical route of apoptosis induction, TRAIL can trigger alternative non-cell death pathways (Figure 3). The induction of NF-κB [65,104,154,160], mitogen-activated protein kinase (MAPK) [161], tyrosine kinase Rous sarcoma oncogene cellular homolog (SRC) and (phosphoinositide 3-kinase (PI3K) pathways [162,163] has been described. TRAIL-induced protein kinase B (AKT) signaling, has been confirmed in numerous cancer entities. AKT is a PI3K-activated protein kinase which is primarily involved in cellular functions like cell growth, survival, and apoptosis. Stimulation of AKT signaling might also play its part in the development of TRAIL-resistance since inhibition of the pathway sensitized initial TRAIL-resistant lung, breast or ovarian cancer cells to TRAIL-induced apoptosis [164,165,166].

Execution of these non-apoptotic, gene-activating pathways is cell-type dependent and occurs when apoptosis induction is prevented or as a result of ‘fractional survival’ in clonal populations of cancer cells. Besides the TRAIL-dependent pro-inflammatory, proliferation, and pro-survival functions of these noted pathways, migration- and invasion-enhancing effects have also been described [159,168,169,170,171,172]. Proliferation-enhancing and migration-promoting effects were found upon administration of exogenous TRAIL [169,171]. These findings enabled the description of a pro-invasive character of endogenous TRAIL in Kirsten rat sarcoma viral oncogene homolog (*KRAS)*-mutated cells. In murine models of *KRAS*-driven precursor lesions and metastasizing PDAC, deletion of mTRAIL-R led to the reduction of tumor growth and metastases, and prolonged mouse survival by constraining cancer cell-autonomous proliferation, migration, and invasion events. Moreover, high TRAIL-R2 expression related to lymph node invasion in human PDAC. Enhanced migration and invasion were attributed to activation of the Rat sarcoma (Ras)-related C3 botulinum toxin substrate 1 (Rac1)/PI3K pathway, mediated by constitutive TRAIL-R2 signals. Therefore, cancer cell-expressed endogenous TRAIL-R2 can promote PDAC throughout different stages. Of note, the signaling pathway was not mediated by the DD of TRAIL-R2, but its membrane-proximal domain (MTD) was necessary [170]. 

The induction of gene-activating pathways by TRAIL is supposed to be communicated via a second cytosolic complex (Figure 3), retaining DISC components FADD and caspase-8 but additionally recruiting receptor-interacting protein kinase 1 (RIPK1), TRAF2 and NF-κB essential modifier (NEMO)/inhibitor of κB (IκB) kinase (IKK) [173,174,175]. Downstream of caspase-8, TRAF2 is essential for cIAP1/2 recruitment, which in turn leads to the ubiquitination of RIPK1 with K11- and K63-linked poly-ubiquitin chains [176]. Ubiquitinated RIPK1 builds the platform for recruitment of linear ubiquitin chain assembly complex (LUBAC), an ubiquitin E3 ligase complex that attaches linear poly-ubiquitin chains on RIPK1 [167]. LUBAC acts in both complexes, alleviating caspase-8 activation and enabling recruitment of the IKK complex, and consequently activation of NF-κB [167,175,177]. 

So far, most known functions of TRAIL receptors are attributed to their presence at the plasma membrane. Nonetheless, high levels of intracellular TRAIL-R1/R2 are characteristic features for many tumors [178,179] and little tangible knowledge is available about the function of repeatedly observed nuclear TRAIL-R2 (nTRAIL-R2) [180,181,182]. Thus, nTRAIL-R2 was found to interact with the Microprocessor complex and its accessory proteins. By this interaction, nTRAIL-R2 negatively regulated the maturation of miRNA let-7, which in turn is a known negative regulator of the expression of various proliferation-driving proteins like high-mobility group AT-hook 2 (HMGA2), Ras and avian myelocytomatosis virus oncogene cellular homolog (c-Myc) [183,184]. As a consequence, by inhibiting let-7-maturation, nTRAIL-R2 enhanced tumor cell proliferation [185]. 

### 1.10. Alternative TRAIL-Mediated Death Signaling 

Besides the induction of non-cell death signaling, TRAIL-Rs have been shown to induce an alternative cell death pathway termed necroptosis reviewed in [186] (Figure 3). Necroptosis is promoted by RIPK1 and RIPK3 [187,188]. In case of inhibited caspase-8 activity, apoptosis induction is prevented and building of the necrosome promoted by interaction of RIPK1 and RIPK3 [189,190]. Necroptosis can be blocked by pharmacological inhibition of the kinase activity of RIPK1 by necrostatin [190,191]. Interestingly, high levels of RIPK1 and RIPK3 have been described for PDAC, as well as their upregulation following treatment with gemcitabine. When the necrosome was blocked, pro-cancer effects have been observed in PDAC cells. Importantly, in vivo no oncogenic progression was detectable when RIP1 or RIP3 were inhibited [192], indicating that alternative anticancer pathways were activated. Deletion of RIPK3 might function in an anti-oncogenic manner via improved peri-tumoral immunogenicity. Since intrinsic resistance against TRAIL-induced apoptosis markedly affects treatment response, the capacity of TRAIL-Rs to mediate further cell-death signaling pathways contributes decisively to the extension of TRAIL-comprising treatment strategies. Particularly, regarding clinical approaches, these discoveries illustrate the urgent priority to gain knowledge on the orchestration of all TRAIL-induced signaling pathways, which might enable the prediction of therapy response and risk evaluation in respect to the promotion of the disease.

## 2. Concluding Remarks and Future Perspectives

Since PDAC is amongst the deadliest of solid tumors, it has become a paradigm for an almost incurable malignancy in today’s research era. PDAC is mostly resistant to conventional therapy. Thus, the discovery of TRAIL created new hope for an alternative anticancer approach. Inducing extrinsic apoptosis, pharmacological targeting of TRAIL-Rs offers the possibility to circumvent resistance against intrinsic apoptosis, a pathway almost invariably inactivated through the loss or mutation of the tumor suppressor p53. However, the majority of primary PDAC cells are resistant or acquire resistance also to TRAIL-induced apoptosis. However, so far apart from promising preclinical studies, with none of the reviewed TRAIL-R agonists a therapeutic advantage in cancer patients has been reported [30]. Presumably, due to inherent TRAIL resistance, the insufficient potency of the used agonist, and a lack of biomarkers for patient stratification, the trials failed to achieve antitumor efficacy. Furthermore, it has emerged that physiological levels of TRAIL can signal so much more than mere apoptosis, which can provoke undesirable effects in the context of anticancer therapy. 

Since tagged variants of recombinant TRAIL-induced hepatotoxicity, the untagged version dulanermin reached clinical testing. The plain fact that hrTRAIL failed to reveal any anticancer activity in various other tumor entities suggests that clinical trials with dulanermin in PDAC would also be unsatisfying. Since TRAIL-R1 was shown to promote apoptosis in PDAC whereas TRAIL-R2 induced pro-inflammatory cytokines, a PDAC trial would be more likely to benefit from a TRAIL-R1-selective treatment [59]. 

Although it is far too early to find a conclusive answer to the question ‘Should we keep walking along the TRAIL for pancreatic cancer treatment?’, several trial options are unexplored, for example TRAIL-R1 selective targeting which surely should be tested. However, selective approaches need to be intensively studied, since recent results demonstrate the dual role of a single TRAIL-R in not only mediating apoptosis but also stimulating non-apoptotic pathways, supporting ‘fractional survival’ [159]. Most importantly, sensitizing strategies to overcome TRAIL resistance [51,193,194] and crosslinked TRAIL-R agonists, ensuring clustering of TRAIL-Rs intensifying DISC formation might be steps towards successful TRAIL therapy. This approach can be enhanced by combination with tagged TRAIL [45,195], to extend half-life period and stability, and to combine TRAIL with agents sensitizing to TRAIL-induced apoptosis [145]. However, restricted drug bioavailability in PDAC caused by reduced perfusion [140] and desmoplastic stroma needs to be conquered. The hopeful outcomes of the in vivo testing of some of the described new TRAIL formulations must be further verified over the next years (i) in a broader spectrum of tumor entities with special attention to mutations and predictive markers, and in (ii) more complex models, representing the heterogeneity and microenvironment of tumors in a manner closer to reality. 

Based on the limited activity of TRAIL-R agonists discussed above, the design of new TRAIL-R agonists aimed at higher potency to induce apoptosis via TRAIL-R1 and TRAIL-R2 [196]. One of these agents, firstly reaching clinical testing, was TAS266, an agonistic tetravalent nanobody directed against TRAIL-R2 [197]. Nanobody approaches can overcome limitations of the efficiency of mAbs in distribution and tumor penetration. Characteristics of variable fragments of Camelid heavy-chain-only antibodies (HcAbs), termed nanobodies, comprise their small size, chemical and thermal stability, high solubility, specificity and affinity, and their simplicity of cloning, as reviewed in [198]. TAS266 is assembled from four identical humanized high-affinity heavy chain domain (VHH) antibody fragments. Since TRAIL-R2 can be engaged by each VHH domain with high affinity, simultaneously clustering of four receptor molecules is possible, therefore intensifying DISC formation. The data obtained revealed that higher valency of DR5 binding correlates with the increased velocity of caspase induction in vitro [197]. However, clinical testing of the substance had to be prematurely terminated, because patients suffered from unexpected symptoms of hepatotoxicity. The underlying mechanisms can only be presumed, including the agent’s immunogenicity and enhanced potency [199]. Therefore, potentially expected immune reactions need to be evaluated extensively preceding de novo clinical testing. 

Efficient cell-death induction by agonistic anti-TRAIL-R antibodies can only be gained due to high clustering capacity. A new class of TRAIL-R agonists possesses this characteristic as it comprises three TRAIL-promoter subsequences united in one polypeptide chain, defined as single-chain-TRAIL-receptor-binding domain (scTRAIL-RBD). Two scTRAIL-RBDs and three receptor binding sites are fused to a protein with hexavalent binding capacity. APG350, the prototype of this approach was created by merging the Fc-part of human IgG1 and the scTRAIL-RBD polypeptide which is thereby able to bind simultaneously six TRAIL-receptors (TRAIL-R1 and TRAIL-R2) per drug molecule. Moreover, APG350 exerts high antitumor potential and induces superior clustering independent of FcγR-mediated cross-linking [200]. Circularly permuted TRAIL (CPT) is a further recombinant version of human TRAIL and is currently clinical investigated for the treatment of hematologic malignancies [201,202,203]. In comparison to wild-type TRAIL, CPT is constructed by the *N*-terminus of amino acid 121–135 sequence of TRAIL and the C-terminus of the amino acid 135–281 sequence of TRAIL connected via a flexible linker. The substance engages TRAIL-R1 and TRAIL-R2 as stable homotrimers and exhibits higher stability and at least improved half-life than wild-type TRAIL [204]. In a single dose-escalation study, no toxicity and maximum tolerated dose was detectable [201]. Depending on the missing preference for one apoptosis-inducing TRAIL-R, the molecule might be effective for PDAC treatment.

Since TRAIL resistance is a severe obstacle, numerous sensitizing strategies in TRAIL-signaling have been envisaged. These may ultimately prevent adverse TRAIL-signaling output on the one hand, and efficiently target PDAC in the future by efficiently inducing apoptosis on the other. Some promising examples of these strategies are described as the substances are already clinically used and are well-described: The herbal anticancer drug paclitaxel enhances TRAIL efficiency for example in gastric cancer cells and prostate cancer stem cells [205,206]. TRAIL-embedded paclitaxel-bound albumin nanoparticles were sufficient in PDAC xenografts [207]. A further plant-derived, cell cycle inhibiting compound, benzyl isothiocyanate (BITC) sensitized *KRAS*-mutated PDAC cells to TRAIL-induced apoptosis [208] and the natural occurring steroidal lactone, withanolide E, and TRAIL cooperate in various human cancer cells by degradation of c-FLIP [209]. Amplification of TRAIL-induced apoptosis was also attributed to the anti-diabetic drug metformin in colorectal cancer cells [210] and to bortezomib by inhibition of the proteasome [211]. Since PDAC is preclinical responsive to histone deacetylase (HDAC) inhibitors [212,213], this approach might also contribute to overcome TRAIL-resistance in pancreatic cancer as shown for multiple myeloma cells and breast cancer cells [214,215]. The intrinsic apoptosis pathway is controlled by the Bcl-2 protein family resulting in cytochrome c release and subsequent activation of downstream caspases. Crosstalk between the two apoptotic pathways can influence cell death induction. The raised expression of anti-apoptotic proteins such as Bcl-2 and Bcl-XL, can constrain the activation of the intrinsic apoptosis pathway and provoke resistance to anti-cancer therapeutics, including TRAIL. Navitoclax (ABT-263) a Bcl-2/Bcl-XL selective inhibitor was reported to sensitize PDAC cells to TRAIL [216]. Moreover, TRAIL resistance can be strikingly overcome by the use of IAP antagonists or SMAC mimetics [217,218] or simultaneous pharmacological inhibition of cyclin-dependent kinase 9 (CDK9), resulting in suppression of anti-apoptotic c-FLIP and Mcl-1 [193]. 

The lack of predictive biomarkers for TRAIL treatment response is likely to be a significant contributing factor that the potential of TRAIL in a convenient clinical setting is not assessable. In a study to identify mediators of TRAIL-sensitivity in various tumor cell lines, among other things in PDAC, the molecule most significantly correlating with response to dulanermin was *N*-acetylgalactosaminyltransferase-14 (GALNT14), responsible for receptor glycosylation and clustering [152]. However, so far, GALNT14 was not clinically proven in pancreatic cancer [219] but might be convenient for prediction of response to death receptor agonists in this setting.

Additionally, intensified knowledge in orchestrated mechanisms of TRAIL-TRAIL-R-signaling, especially in *KRAS*-mutated PDAC [170], would present a further approach towards targeted therapy. Since the endogenous TRAIL-TRAIL-R-system promotes *KRAS*-mutated tumor growth, invasion and metastases in PDAC, future therapeutic strategies seeking to harness TRAIL’s apoptosis-inducing activity should aim to block this unwanted pro-tumorigenic effect and in turn boost TRAIL-induced pro-apoptotic pathways.

## Abbreviations

5-FU	5-fluorouracil
ADCC	antibody-dependent cellular cytotoxicity
AKT	protein kinase B
ATM	mutated in the disease ataxia telangiectasia
ATP	adenosine triphosphate
Apo2L	Apo-2 ligand
Bak	Bcl-2 antagonist or killer
Bax	Bcl-2-associated X protein
Bcl-2	B-cell CLL/lymphoma 2
Bcl-XL	B-cell lymphoma-extra-large
BH3	Bcl-2 homology domain 3
Bid	BH3-only protein
BITC	benzyl isothiocyanate
CDK9	cyclin-dependent kinase 9
c-FLIP	cellular (FADD-like IL-1β-converting enzyme)-inhibitory protein
cIAP	cellular IAP
c-Myc	avian myelocytomatosis virus oncogene cellular homolog
DcR	decoy receptor
CPT	circularly permuted TRAIL
DD	death domain
DDR	DNA damage response
DGCR8	DiGeorge critical region8
DR	death receptor
ER	endoplasmic reticulum
ERK	extracellular signal-regulated kinase
FADD	Fas-associated death domain
Fc	Fragment crystallizable
Flag	DYKDDDDK
FLICE	FADD-like IL-1β-converting enzyme
FOLFIRINOX	FOL-folinic acid, F-5-fluorouracil (5-FU), IRIN-irinotecan, OX-oxaliplatin
GALNT14	*N*-acetylgalactosaminyltransferase-14
GTP	guanosine-5′-triphosphate
HcAbs	heavy-chain-only antibodies
HDAC	histone deacetylase
HMGA2	high-mobility group AT-hook 2
IAP	inhibitor of apoptosis protein
IGF-R	insulin-like growth factor receptor
IgG	immunoglobulin
IGFR	insulin-like growth factor receptor
IKK	inhibitor of κB kinase
IMS	intermembrane space
IκB	inhibitor of κB
IZ	isoleucine zipper
JNK	c-JUN *N*-terminal kinase
KRAS	Kirsten rat sarcoma viral oncogene homolog
LUBAC	linear ubiquitin chain assembly complex
LZ	leucine zipper
mAb	monoclonal antibody
MAPK	mitogen-activated protein kinase
Mcl-1	myeloid leukemia cell differentiation protein
memTRAIL	membrane-bound TRAIL
miRNA	micro RNA
MOM	mitochondrial outer membrane
MOMP	mitochondrial outer membrane permeabilization
MTD	membrane-proximal domain
nab	nanoparticle albumin-bound
NEMO	NF-κB essential modifier
NF-κB	nuclear factor kappa light chain enhancer of activated B cells
*N*-glyc	*N*-glycosylation
NIK	NF-κB-inducing kinase
nTRAIL	nuclear TRAIL
*O*-glyc	*O*-glycosylation
OPG	osteoprotegerin
p38 MAPK	p38 mitogen-activated protein kinase
PARP	poly-ADP ribose polymerase
PCD	programmed cell death
PDAC	pancreatic ductal adenocarcinoma
Rac1	Ras-related C3 botulinum toxin substrate 1
RANKL	receptor activator of nuclear factor κB ligand
Ras	Rat sarcoma
rhTRAIL	recombinant human TRAIL
RIPK	receptor-interacting serine/threonine protein kinase 1
scTRAIL-RBD	single-chain-TRAIL-receptor-binding domain
Smac/DIABLO	second mitochondrial activator of caspases/direct inhibitor of apoptosis-binding protein with low pI
SR	superfamily
SRC	Rous sarcoma oncogene cellular homolog
STAT	signal transducer and activator of transcription
sTRAIL	soluble TRAIL
TNF	tumor necrosis factor
TAPE	threonine, alanine, proline, glutamine
TRAIL	TNF-related apoptosis-inducing ligand
TRAIL-R	Trail receptor
Ub	ubiquitin
VHH	heavy chain domain
XIAP	X-linked inhibitor of apoptosis protein
